# Reviewed article: Azevedo SPF, Damião JJ, Castro IRR. Brazilian dietary guidelines: interfaces with the mother and child hospital context. Epidemiol Serv Saude. 2025:34;e20240391.

**DOI:** 10.1590/S2237-96222024v34e20240391.b

**Published:** 2025-05-02

**Authors:** Renata Adrielle Lima Vieira

**Affiliations:** Universidade Federal da Paraíba Departamento de Nutrição, Departamento de Nutrição

**Table:** 

Rating	Excellent	Good	Average	Below average	Poor
Interest	✓				
Quality				✓	
Originality	✓				
Overall		✓			

**Table:** 

Questionnaire	Yes	No	Not applicable
Does the manuscript contain new and significant information to justify publication?	✓		
Does the Abstract (Summary) clearly and accurately describe the content of the article?	✓		
Is the problem significant and concisely stated?	✓		
Are the methods described comprehensively?	✓		
Are the interpretations and conclusions justified by the results?	✓		
Is adequate reference made to other work in the field?	✓		

## Manuscript Structure:

Length of article is: Adequate

Number of tables is: Adequate

Number of figures is: Adequate

Please state any conflict(s) of interest that you have in relation to the review of this paper (state “none” if this is not applicable): None

## Comments

O manuscrito apresenta o desenvolvimento de uma matriz que estabelece conexões entre os princípios e recomendações dos Guias Alimentares Brasileiros e as ações de cuidado alimentar e nutricional realizadas no contexto hospitalar, especificamente em um hospital materno-infantil. Trata-se de um estudo teórico-conceitual que mapeia espaços de cuidado no hospital, como unidades de internação e o banco de leite humano, identificando oportunidades para aplicar os guias alimentares nesses ambientes.

Contudo, alguns ajustes são necessários:

1-Resumo: estruturado, claro, com os devidos elementos necessários. No entanto, as palavras chaves: cuidado nutricional e painel de especialistas não foram encontradas no DeCS. Trocá-las.

2- Introdução: possui apenas 2 parágrafos, apresentando algumas lacunas importantes que comprometem a contextualização adequada do problema abordado. Embora destaque a importância dos Guias Alimentares e seu papel nas políticas públicas, falta uma explicação mais detalhada sobre a relevância específica da aplicação desses guias no ambiente hospitalar, especialmente no contexto materno-infantil. Será necessário incluir mais informações sobre o impacto da alimentação adequada em pacientes hospitalizados e o papel crítico que os hospitais desempenham na promoção de práticas alimentares saudáveis. Esses pontos ajudariam a justificar de maneira mais sólida a importância do estudo e a lacuna que ele se propõe a preencher.

3-Métodos: A escolha de um local de trabalho de uma das autoras como justificativa para o desenvolvimento de um projeto não é considerada plausível, pois não reflete critérios científicos, metodológicos ou estratégicos. A seleção do local deve ser fundamentada em aspectos como a relevância da população-alvo, a disponibilidade de recursos necessários para a pesquisa ou a viabilidade de implementação das intervenções propostas, garantindo que a escolha esteja alinhada com os objetivos e hipóteses do estudo. Sugiro retirar esse trecho e melhor o tópico contexto.

No trecho: “Bases teóricas e conceituais: foram adotados como referenciais teóricos os Guias Alimentares Brasileiros(3,4).” Deixar escrito e claro o título completo dos guias como é abordado em outros trechos do manuscrito.

Além disso, deixar claro e justificado que foi utilizado “como base o modelo que analisou as interfaces entre o Guia da População Brasileira com a prática de nutricionistas na área de alimentação coletiva(12).” por falta de modelos no contexto hospitalar geral.

“Foram excluídos: unidades de terapia intensiva e de pacientes graves e a enfermaria de Doenças Infecciosas Pediátricas - pela restrição de acesso a esses locais;” . A exclusão das unidades de terapia intensiva (UTI) e da enfermaria de Doenças Infecciosas Pediátricas foi justificada pela restrição de acesso a esses locais. No entanto, acredito que a exclusão da UTI deva ser fundamentada não apenas na restrição de acesso, mas também pela especificidade do tratamento nutricional empregado nesse ambiente, que envolve terapias de alta complexidade, como o uso de fórmulas infantis, nutrição enteral e parenteral. Isso diferencia o contexto da UTI em relação à promoção da saúde nutricional, tornando o enfoque distinto. Sugiro, portanto, que a justificativa para a exclusão da UTI seja aprimorada, considerando esses aspectos técnicos.

No tópico “Etapas do estudo:” está extenso. Sugiro fazer um parágrafo a partir do trecho “A terceira etapa consistiu na identificação das potenciais interfaces entre as ações de cuidado alimentar e nutricional (desenvolvidas ou por desenvolver) nos espaços selecionados e os trechos dos Guias, e posterior sistematização dessas interfaces”

No mesmo tópico “Etapas do estudo” contem que a etapa 3 foi organizada em 5 sub etapas. No entanto, apenas quatro sub etapas são relatadas, ou acrescentar a sub etapa faltante ou ajustar no texto que são 4 e não 5.

4- Resultados: padronizar a nomenclatura Guia Alimentar para Crianças Brasileiras Menores de Dois Anos ao invés de Guia da Criança.

O primeiro parágrafo está longo, o que pode comprometer a clareza e a fluidez da leitura. Sugiro que o texto seja segmentado em partes menores.

Quadro 1- corrigir separação da palavra “outros” no item unidades de internação e ambulatórios ; “consideração” no item cozinha dietética

